# The Hospital Laundry

**Published:** 1916-04-22

**Authors:** 


### THE HOSPITAL LAUNDRY.

### I.—Its Constituent Parts, Equipment, and Staff.

A hospital of over fifty beds cannot be considered to be fully equipped unless it can number a steam-power laundry amongst its departments. If the machinery is suitable and in good order the cost of working is considerably less than that of sending the linen to a public laundry; less stock is required in the wards, as it is returned more quickly; there is not so much likelihood of it being lost; and it is besides a great convenience for the matron to have this important service of clothing and linen under her direct control.

The structure for a laundry of 120 beds will probably comprise: (1) A sorting-room with low divisions for separating the different kinds of articles; (2) a wash-house; (3) an ironing-room; (4) a small office; (5) a delivery-room with wooden pigeon-holes for sorting the staff laundry. The engine-room does not come within the scope of this article; it is only entered by the engineer or a man working under his orders, who is also responsible for the furnace, boilers, and mechanical parts of the machines. It is not necessary for any other men to be employed in the laundry, and it is best to employ only women. The equipment of the wash-house consists of two washing machines, to which may be added a foul-washing machine. The two ordinary ones should be 250-shirt size and 100-shirt size respectively. The smaller one is used daily for theatre and babies' washing, and on washing days both are in use. Four tanks, including two for blueing and starching, to which wringer is attached, washing troughs, soap boiler, and a hydro extractor. The ironing-room contains a large four-roller ironing machine or calender. This machine fulfils all the requirements of ironer and mangle, practically everything can be ironed, sheets, table linen, even towels, table napkins, shirts, etc.; nurses' aprons, if the skirts are gored and not gathered, can also be partially ironed by it, but will need finishing by hand. Six gas-irons, including one Jumbo iron for skirts of nurses' dresses, one collar and cuff machine with an adjustment for shirt-fronts, one goffering machine, and two ironing horses complete the equipment of the ironing-room. The drying closet is approached from the ironing-room, and contains six iron horses on runners, resembling gigantic towel horses. They must not be confused with the ironing horses, which are posts with many arms on which are hung freshly ironed garments which must not be crushed. There are, of course, ironing tables, skirt and sleeve boards, a chair for each worker, three wooden trolleys for conveying the clothes from room to room, six large hampers on wheels for conveying clothes to the wards; these must on no account be used for soiled linen, and must be hosed and scrubbed frequently. Foot-boards just large enough for one person to stand on should be provided for the washhouse, so that persons entering on some errand can step from one to another and thus avoid getting wet feet. They must be small, in order to allow the trolleys to pass beside them.

### The Staff.

The laundry staff should live in the hospital premises, and should not be natives of the town. The risk of articles disappearing is not great if these two rules can be observed. In a small laundry such as is being described it is better to engage a capable woman, who has been forewoman in a commercial laundry and thoroughly understands the book work and the machines, as head laundress than to put the laundry under the charge of a lady who has not had as much practical experience. She must have a comfortable bedroom and will take her meals with the upper servants; her wages will be £30 a year and uniform. Four laundry maids will work under her orders, their wages ranging from £12 to £24 a year with uniform. It is a good plan to arrange for the two younger maids to do housework during the early hours of the morning and come to the laundry at 9 a.m. for the rest of the day.

The hours of duty are 7 a.m. to 6 p.m., with the usual intervals for meals, which are partaken of in the servants' hall.

[Part II. will deal with the week's work in the laundry.]

